# Reliable Automated Displacement Monitoring Using Robotic Total Station Assisted by a Fixed-Length Track

**DOI:** 10.3390/s26020746

**Published:** 2026-01-22

**Authors:** Yunhui Jiang, He Gao, Jianguo Zhou

**Affiliations:** 1 China Institute of Water Resources and Hydropower Research, Beijing 100038, China; 2 School of Civil Engineering, Architecture and Environment, Hubei University of Technology, Wuhan 430068, China

**Keywords:** automation, displacement measurement, monitoring system, robotic total station

## Abstract

Robotic total stations are multi-sensor integrated instruments widely used in displacement monitoring. The principles of polar coordinate or forward intersection systems are usually utilized for calculating monitoring results. However, the polar coordinate method lacks redundant observations, leading to unreliable results sometimes. Forward intersection requires two instruments for automated monitoring, doubling the cost. In this regard, this paper proposes a novel automated displacement monitoring method using the robotic total station assisted by a fixed-length track. By setting up two station points at both ends of a fixed-length track, the robotic total station is driven to move back and forth on the track and obtain observations at both station points. Then, automated monitoring based on the principle of forward intersection with a single robotic total station is achieved. Simulation and feasibility tests show that the overall accuracy of forward intersection is better than that of polar coordinate system as the monitoring distance increases. At the same time, regardless of tracking a prism or not, the robotic total station is able to automatically find and aim at the targets when moving between station points on the track. Further practical tests show that the reliability of the monitoring results of the proposed method is superior to the polar coordinate method, which provides new ideas for ensuring the reliability of results while reducing cost in actual monitoring tasks.

## 1. Introduction

As a typical multi-sensor-integrated instrument, a robotic total station is developed from a conventional manual total station [1,2]. Besides the basic angle encoder and electronic distance measurement sensor for accurate angular and distance sensing, its capabilities have been greatly expanded with the addition of modules like a servomotor, imaging sensor, etc. The servomotor moves the telescope of the robotic total station horizontally and vertically to the approximate position of the target, which is usually a prism with high reflectivity. The accurate position of the prism can be determined by obtaining the maximum of the strength of the laser-beam reflected, which is called automatic target recognition (ATR) [3]. In this way, multiple targets can be positioned in sequence. Moreover, the larger field of view provided by the imaging sensor makes it possible for the robotic total station to track a moving prism automatically, as the shift indicated by the imaging sensor can be computed [4] even when contact with the prism is lost temporarily.

The capabilities of a robotic total station, including detecting, recognizing, aiming, and tracking a prism automatically, increase the accuracy and productivity of its applications [5,6,7]. For traditional surveying applications, Y. Liang et al. [8] proved the effectiveness of establishing a high-precision measurement control field with the robotic total station. F. Zhu et al. [9] proposed a highly accurate and efficient railway track irregularity measuring system utilizing the robotic total station constraint. G. Bitelli et al. [10] presented a method for determining height differences when crossing impassable areas with the robotic total station. J. Zhou et al. [11] developed the height difference determination method with robotic total stations assisted by unmanned aerial vehicles (UAVs). J. Liu et al. [12] developed a new method to increase the accuracy of a gun barrel using a robotic total station, which showed higher accuracy than other related conventional methods. Machine guidance is another important application of a robotic total station, utilizing its capability of tracking. Combined with other sensors, the robotic total station is used to track the 360° prism installed on a platform-cart to monitor lift-thickness during highway construction [13]. Kinematic positioning with a robotic total station is applied to UAVs to improve the positioning accuracy, especially in global navigation satellite system (GNSS)-denied environments [14,15]. The tracking function of the robotic total station is combined with photographic imaging to achieve real-time dynamic coordinate measurement for double-shield tunnel boring machine guidance [16]. Displacement monitoring of civil infrastructures is one of the crucial tasks for ensuring structural safety [17]. The capability of obtaining accurate 3D coordinates of multiple targets in sequence has afforded the robotic total station an important role in the continuous and automated displacement monitoring of civil infrastructures and natural hazards in recent years [18,19,20,21]. Usually, the robotic total station is placed on a forced-centering pillar in the measurement chamber to repeat observations with the required frequency to detect potential displacements of monitoring points and provide early warning. Y. B. Luo et al. [22,23] presented the calculation formulas for the crown settlement, wall convergence, and horizontal displacement of a tunnel by a robotic total station, and tests showed that measurement of displacement using the robotic total station is more convenient than that by traditional methods. For dams, the robotic total station, integrated with other instruments and sensors, provides solutions for measuring 3D displacements of the dam body and slopes on both banks with an improved temporal and spatial resolution [24,25,26]. Merging the data acquired by the robotic total station, the camera, and the laser scanner, the spatially distributed displacements of landslides are monitored, providing a widely applicable tool for revealing the complex dynamic nature of these events [27,28].

Current automated displacement monitoring systems using a robotic total station are mainly based on the principles of polar coordinate or forward intersection methods [29]. For the polar coordinate system, the coordinates of monitoring points are calculated with angular and distance observations from one station point. Although the implementation of the system is simple, as only one instrument is required, the reliability of monitoring results with the polar coordinate system cannot be guaranteed due to the lack of redundant observations, especially when affected by complex meteorological conditions. Automated monitoring with forward intersection usually requires the participation of two robotic total stations from two station points. The monitoring results obtained from the combination of two instruments are more reliable compared to those of the polar coordinate system, but the hardware cost is doubled. Therefore, the task of reducing cost while maintaining reliability for a robotic total station-based monitoring system deserves great attention. Inspired by ground-based synthetic aperture radar instruments [30], this paper proposes an automated displacement monitoring method using a robotic total station assisted by a fixed-length track. By setting two station points at both ends of a fixed-length track, the robotic total station moves in between them, driven by a motor, and conducts observations. With the observations obtained at both station points, automated monitoring with forward intersection is implemented with a single robotic total station.

The remaining sections of this article are organized as follows. Section 2 explains the mathematical principles and system components of the proposed method. Section 3 describes the experiments, including simulations, feasibility, and practical tests first, and discusses and analyzes the results then. Section 4 is dedicated to conclusions and research prospects.

## 2. Methods

To address the issue of current monitoring methods based on polar coordinate or forward intersection systems, a novel automated displacement monitoring method using one robotic total station assisted by a fixed-length track is proposed. Initially, a fixed-length track is precisely installed to ensure that the robotic total station moves back and forth on the track horizontally, and two station points are established at each end of the track. Then, the robotic total station moves to one station point to perform manual training; that is, all the monitoring points are sighted by the robotic total station manually, and corresponding angular and distance observations are recorded and stored to remember the positions of these points. The training data at another station point can be computed automatically. Finally, the robotic total station is driven by the motor to move between the station points at two ends of the fixed-length track, obtaining observations at each monitoring point based on the training data. These observations are processed based on the principle of forward intersection to compute the coordinates and displacements of the monitoring points.

### 2.1. Mathematical Principles

#### 2.1.1. Manual Training and Automated Monitoring

**Figure 1:**
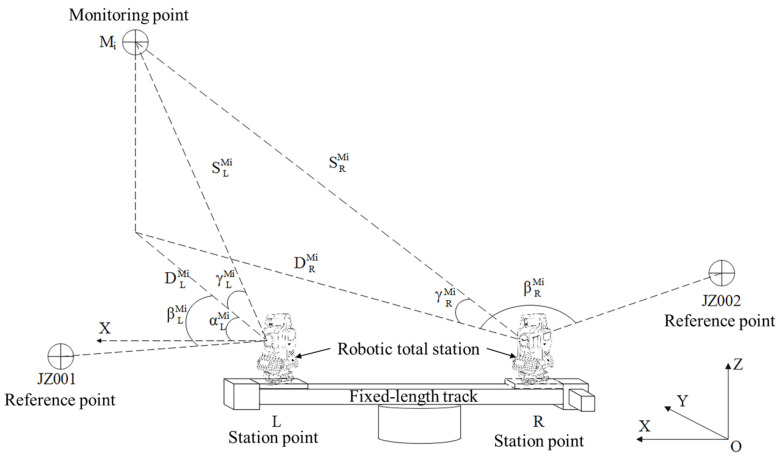
Principle of manual training for the proposed method.

The fundamental principle of manual training for the proposed method is illustrated in Figure 1. Station points, defined as L and R, are located at the ends of the fixed-length track. Two reference points, JZ001 and JZ002, are used as the reference directions for L and R, respectively. The known 3D coordinates of L and R are defined as $\left(X_{L},Y_{L},Z_{L}\right)$ and $\left(X_{R},Y_{R},Z_{R}\right)$. The coordinates of JZ001 and JZ002 are represented by $\left(X_{J1},Y_{J1},Z_{J1}\right)$ and $\left(X_{J2},Y_{J2},Z_{J2}\right)$. Initially, the robotic total station is leveled at L with measured instrument height i. Then, it is rotated to sight JZ001 for orientation, and the corresponding slope distance $S_{L}^{J}$, horizontal angle reading $\beta_{L}^{J}$, and vertical angle reading $\gamma_{L}^{J}$ are recorded. Finally, the robotic total station is rotated to the sight monitoring point $M_{i}$, and records the observed slope distance $S_{L}^{M_{i}}$, horizontal angle reading $\beta_{L}^{M_{i}}$, and vertical angle reading $\gamma_{L}^{M_{i}}$. Meanwhile, the coordinates of $M_{i}$ can be calculated based on the principle of the polar coordinate system.

The azimuth $\alpha_{L}^{J}$ between L and JZ001 can be calculated with their known coordinates. Then, the azimuth $\alpha_{L}^{M_{i}}$ between L and the $M_{i}$ can be derived as

(1) $$\alpha_{L}^{M_{i}}=\alpha_{L}^{J}+(\beta_{L}^{M_{i}}-\beta_{L}^{J})$$

The coordinates of $M_{i}$, denoted as $\left(X^{M_{i}},Y^{M_{i}},Z^{M_{i}}\right)$, are computed as

(2) $$\left\{\begin{array}{l} X^{M_{i}}=X_{L}+S_{L}^{M_{i}}*\cos\gamma_{L}^{M_{i}}*\cos\alpha_{L}^{M_{i}}\\ Y^{M_{i}}=Y_{L}+S_{L}^{M_{i}}*\cos\gamma_{L}^{M_{i}}*\sin\alpha_{L}^{M_{i}}\\ Z^{M_{i}}=Z_{L}+S_{L}^{M_{i}}*\sin\gamma_{L}^{M_{i}}+i-v_{M_{i}} \end{array}\right.$$

Using the computed coordinates of the monitoring points, when the robotic total station moves to the station point R, the training data can be automatically calculated, which minimizes labor costs and improves automation. As shown in Figure 1, the training data $S_{R}^{M_{i}}$, $\beta_{R}^{M_{i}}$, and $\gamma_{R}^{M_{i}}$ of $M_{i}$ for the robotic total station at R can be obtained using the following formulas:

(3) $$\left\{\begin{array}{l} S_{R}^{M_{i}}=\sqrt{{\left\{\left(Z_{R}+i\right)-(Z^{M_{i}}+v_{M_{i}})\right\}}^{2}+{\left(X^{M_{i}}-X_{R}\right)}^{2}+{\left(Y^{M_{i}}-Y_{R}\right)}^{2}}\\ \beta_{R}^{M_{i}}=\arctan\frac{X^{M_{i}}-X_{R}}{Y^{M_{i}}-Y_{R}}-\arctan\frac{X_{J2}-X_{R}}{Y_{J2}-Y_{R}}\\ \gamma_{R}^{M_{i}}=\arcsin\frac{(Z^{M_{i}}+v_{M_{i}})-\left(Z_{R}+i\right)}{\sqrt{{\left\{\left(Z_{R}+i\right)-(Z^{M_{i}}+v_{M_{i}})\right\}}^{2}+{\left(X^{M_{i}}-X_{R}\right)}^{2}+{\left(Y^{M_{i}}-Y_{R}\right)}^{2}}} \end{array}\right.$$

$S_{R}^{M_{i}}$ can be used to verify whether the monitoring point has been correctly sighted during the automated monitoring.

After obtaining the training data for the monitoring points, automated monitoring can be performed according to the required frequency. Considering the differences in the functions of robotic total stations, when the robotic total station can track the prism, it completes the automated monitoring at the station point on one end of the track first, for example L, based on the training data. Then it tracks the last monitoring prism and moves to the other end of the track. The automated orientation at R can be achieved by utilizing the coordinates of R and the tracked monitoring point. Finally, automated monitoring can be carried out at R based on the training data. When the robotic total station does not provide the function of tracking, the azimuth $\theta_{L}$ of the last monitoring point at L is recorded. The robotic total station moves to R while maintaining the telescope orientation of the robotic total station after completing the monitoring of the last point at L. Based on the coordinates of R and the corresponding reference point JZ002, the azimuth $\theta_{R}$ between them can be calculated. Then the horizontal angle μ at which the telescope of the robotic total station needs to rotate from its current position to JZ002 can be calculated as

(4) $$\mu =\theta_{R}-\theta_{L}$$

Finally, the robotic total station completes the orientation based on μ and the vertical angle towards JZ002, and automated monitoring can be conducted based on the training data at R.

#### 2.1.2. Calculation of Coordinates of Monitoring Points

After obtaining the observations for monitoring point $M_{i}$ from both station points at each monitoring epoch, the principle of forward intersection can be utilized to calculate the coordinates of $M_{i}$. Similarly to Figure 1, $\beta_{L}^{M_{i}}$ and $\beta_{R}^{M_{i}}$ are horizontal angle observations from L and R, and $D_{L}^{M_{i}}$ and $D_{R}^{M_{i}}$ represent the horizontal distance observations. As $\beta_{L}^{M_{i}}$ and $\beta_{R}^{M_{i}}$ come from the same instrument, they have the same mean square error $m_{\beta }$, which is set as the unit weight. $m_{D_{L}^{M_{i}}}$ and $m_{D_{R}^{M_{i}}}$ denote the mean square errors of $D_{L}^{M_{i}}$ and $D_{R}^{M_{i}}$. As $D_{L}^{M_{i}}$ and $D_{R}^{M_{i}}$ are much larger than the distance between the two station points, it can be considered that $D_{L}^{M_{i}}\approx D_{R}^{M_{i}}$ and $m_{D_{L}^{M_{i}}}\approx m_{D_{R}^{M_{i}}}=m_{D}$. Then the weights of observations are

(5) $$P_{\beta_{L}^{M_{i}}}=P_{\beta_{R}^{M_{i}}}=1;P_{D_{L}^{M_{i}}}=P_{D_{R}^{M_{i}}}=\frac{m_{\beta }^{2}}{m_{D}^{2}}$$

Based on the principle of least squares adjustment, the error equations of forward intersection can be constructed as follows:

(6) $$\left\{\begin{array}{l} \nu_{D_{L}^{M_{i}}}=\cos\alpha_{L}^{0}dx_{M_{i}}+\sin\alpha_{L}^{0}dy_{M_{i}}+l_{D_{L}^{M_{i}}}\\ \nu_{D_{R}^{M_{i}}}=\cos\alpha_{R}^{0}dx_{M_{i}}+\sin\alpha_{R}^{0}dy_{M_{i}}+l_{D_{R}^{M_{i}}}\\ \nu_{\beta_{L}^{M_{i}}}=\frac{\rho }{D_{L}^{0}}\cos\alpha_{L}^{0}dx_{M_{i}}-\frac{\rho }{D_{L}^{0}}\cos\alpha_{L}^{0}dy_{M_{i}}+l_{\beta_{L}^{M_{i}}}\\ \nu_{\beta_{R}^{M_{i}}}=\frac{\rho }{D_{R}^{0}}\cos\alpha_{R}^{0}dx_{M_{i}}-\frac{\rho }{D_{R}^{0}}\cos\alpha_{R}^{0}dy_{M_{i}}+l_{\beta_{R}^{M_{i}}} \end{array}\right.$$

where $\nu_{D_{L}^{M_{i}}}$,$\nu_{D_{R}^{M_{i}}}$,$\nu_{\beta_{L}^{M_{i}}}$, and $\nu_{\beta_{R}^{M_{i}}}$ represent the corrections for observations. $l_{D_{L}^{M_{i}}}=D_{L}^{0}-D_{L}^{M_{i}},l_{D_{R}^{M_{i}}}=D_{R}^{0}-D_{R}^{M_{i}}$, $l_{\beta_{L}^{M_{i}}}=\beta_{L}^{0}-\beta_{L}^{M_{i}},l_{\beta_{R}^{M_{i}}}=\beta_{R}^{0}-\beta_{R}^{M_{i}}$, $\rho =206265^{\prime \prime }$, $D_{L}^{0},D_{R}^{0},\beta_{L}^{0},\beta_{R}^{0}$ are the approximate observations; $\alpha_{L}^{0},\alpha_{R}^{0}$ are the approximate azimuths from both station points to the monitoring point. All of them can be computed using the known coordinates of L, R and the approximate coordinates $\left(X_{M_{i}}^{0},Y_{M_{i}}^{0}\right)$ of $M_{i}$. The error equations can be written in matrix form as follows:

(7) $$\nu =Β\hat{x}-l$$

The corrections $dx_{M_{i}}$ and $dy_{M_{i}}$ for the coordinates of $M_{i}$ can be calculated as

(8) $$\hat{x}=\left[\begin{array}{c} dx_{M_{i}}\\ dy_{M_{i}} \end{array}\right]={({Β}^{Τ}PB)}^{-1}B^{Τ}Pl$$

where ν is the correction matrix of observations, Β is the coefficient matrix, l is the constant matrix, and P is the weight matrix.

The adjusted coordinates of $M_{i}$ can be computed as

(9) $$\begin{array}{l} {\hat{X}}_{M_{i}}=X_{M_{i}}^{0}+dx_{M_{i}}\\ {\hat{Y}}_{M_{i}}=Y_{M_{i}}^{0}+dy_{M_{i}} \end{array}$$

Compared with the traditional forward intersection method, which requires two instruments, many system error sources can be reduced or eliminated using the single-instrument track-based method. Firstly, the track-based method requires only one robotic total station, which avoids inter-instrument system errors. Secondly, the coordinates of two station points for the traditional method are generally obtained through control network adjustment, and the accuracy is not as good as the track-based method. Lastly, the two station points are usually located far away from each other for the dual-instrument method, resulting in different meteorological effects on observations. Instead, the two station points of the track-based method are adjacent, then meteorological effects on observations are consistent.

### 2.2. System Components

**Figure 2:**
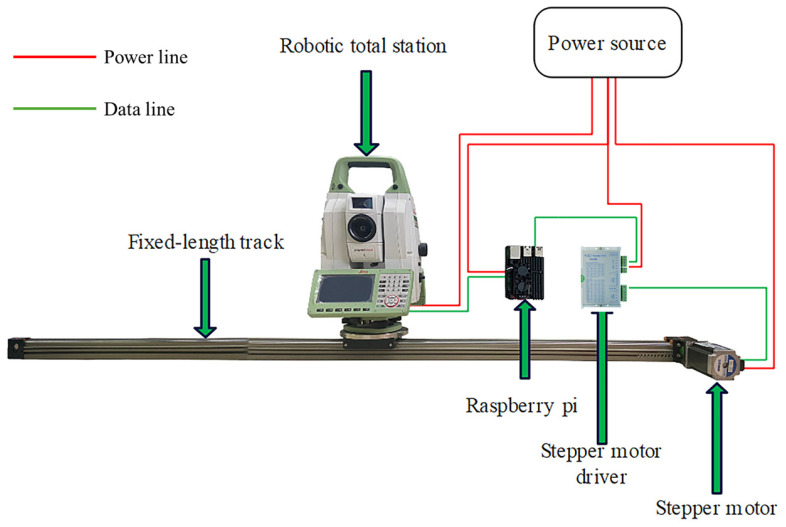
The system components of the automated monitoring system.

The automated robotic total station monitoring system, depicted in Figure 2, is primarily composed of three components: the control system, the track system, and the robotic total station. These components cooperate with each other to achieve unmanned automated monitoring by moving the robotic total station along the fixed-length track.

The control system is composed of a Raspberry Pi, which is a miniature computer based on the Linux operating system. Despite its credit card-sized form factor, Raspberry Pi possesses the majority of fundamental personal computer functionalities. Raspberry Pi allows users to engage in data exchange with hardware devices through GPIO interfaces, fulfilling specific requirements. Software developed with Python 3.10.9 for both the track and robotic total station is stored in the control system. Its primary role involves governing the movement of the robotic total station along the track, obtaining observations, and conducting data processing tasks.

The track system comprises a fixed-length track, a stepper motor, and a stepper motor driver. Raspberry Pi utilizes its internal GPIO interfaces to issue commands to the stepper motor driver, prompting the stepper motor driver to provide pulse signals to the stepper motor. Consequently, the stepper motor translates these pulse signals into corresponding angular displacements, thereby facilitating the unrestricted movement of the robotic total station fixed on the sliding platform along the fixed-length track.

The basic functions of the robotic total station are to provide angular and distance observations towards multiple targets. Connected to the control system through the serial port, manual training and automated monitoring can be achieved. Manual training is a crucial prerequisite for automated monitoring, which means that the robotic total station is driven to locate the monitoring points and obtain the observations automatically using the positions remembered during the training phase with predefined monitoring frequency. The observations obtained during each monitoring epoch are processed using a least squares algorithm to get the monitoring results.

For the cost of the system, the main expense is the robotic total station. Taking the Leica TM50 robotic total station, commonly used for monitoring, as an example, its market price is about USD 50,000, while Raspberry Pi and the track system generally only cost a few hundred dollars. Compared with the traditional forward intersection monitoring system, which requires two robotic total stations, the cost of the proposed system is almost halved.

For practical engineering deployment, the resistance to deformation and the wear quality of the materials used for manufacturing tracks should be considered. And it is suggested to use an Invar alloy with extremely low thermal expansion coefficients. When installing the track, it is necessary to ensure its leveling, especially to ensure that the leveling of the robotic total station is within the scope of the instrument tilt automatic compensator when it moves to station points L and R. If necessary, this can be achieved through an automatic leveling base. To ensure the accuracy of station points coordinates, the distance between station points L and R on the track can be accurately calibrated after installation, and limited switches can be installed on the track to control the range of motion of the stepper motor and improve accuracy.

## 3. Experiments and Results

### 3.1. Simulations

Although forward intersection conditions are met by moving the robotic total station to two station points located at both ends of the track for observation, the distance between the two station points is quite short due to the limited track length. Then the accuracy between the forward intersection under such a situation and the polar coordinate system is compared through simulations.

**Figure 3:**
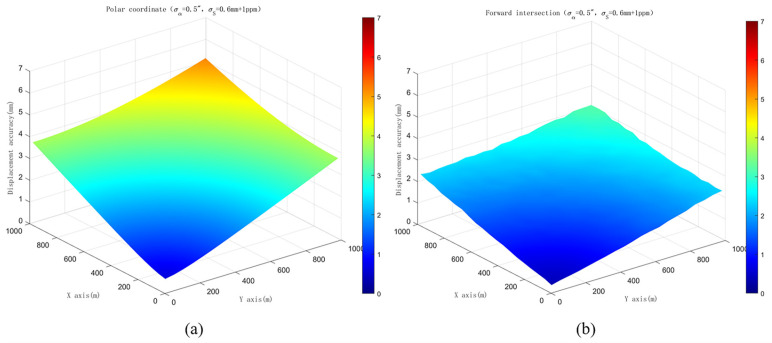
Accuracy comparison with simulations: (**a**) polar coordinate; (**b**) forward intersection.

Taking only the accuracy of the robotic total station into consideration, the simulation is conducted with the robotic total station commonly used in displacement monitoring, Leica TM50, which has a nominal angle measurement accuracy of 0.5″ and a distance measurement accuracy of 0.6 mm + 1 ppm. For the polar coordinate system, the simulation scenario is set with coordinates of the station point as (0, 0). As for the forward intersection, the coordinates of the two station points are set as (0, −1) and (0, 1), respectively, resulting in a distance of 2 m between the two stations. Considering the symmetry, the mean square errors of monitoring points within a 1000 m range in the first quadrant are calculated using both polar coordinate and forward intersection methods. The results are shown in Figure 3. Figure 3a represents the mean square errors of monitoring points calculated using the polar coordinate system. It can be observed from the figure that the errors of the monitoring points increase with the distances from the station point to the monitoring points for the polar coordinate system. Figure 3b shows the results obtained using forward intersection based on the observations from the two station points. Compared with the polar coordinate system, the accuracy differences in monitoring points are not obvious within a 400 m range. As the monitoring distance further increases, the difference between the two methods gradually becomes apparent, and the accuracy of the monitoring points obtained through forward intersection is always superior to that of the polar coordinate system. In the simulation scenario, the maximum mean square error of monitoring points based on the polar coordinate system exceeds 5 mm, while that based on forward intersection remains at around 3 mm. The simulation results verify that, even with a distance of only 2 m between the two station points, the performance of the forward intersection is still better than the polar coordinate system, which provides a theoretical basis for utilizing a single robotic total station to achieve reliable displacement monitoring.

**Figure 4:**
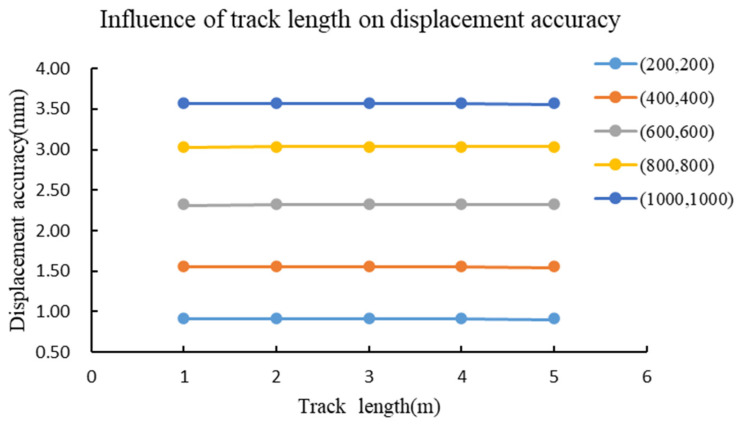
The influence of track length on displacement accuracy.

To further quantify the impact of track length, or the distance between two station points, on the accuracy of forward intersection, simulations are conducted. The track length is specifically set to vary between 1 and 5 m at a 1 m interval. Five typical monitoring points, including (200, 200), (400, 400), (600, 600), (800, 800), and (1000, 1000), are selected to analyze displacement accuracy under different track lengths. The results are shown in Figure 4. As illustrated, the displacement errors of monitoring points increase with their distance from the track. However, for a specific monitoring point, changes in track length have a minimal impact on displacement accuracy. For the convenience of transportation and on-site installation, the track length can be set to around 2 m.

### 3.2. Feasibility Tests

To verify the feasibility of the proposed method, experiments are conducted to analyze the impact of system errors from different instruments, the differences in the polar coordinate system, resulting from different station points, and the differences between the polar coordinate and forward intersection systems.

**Figure 5:**
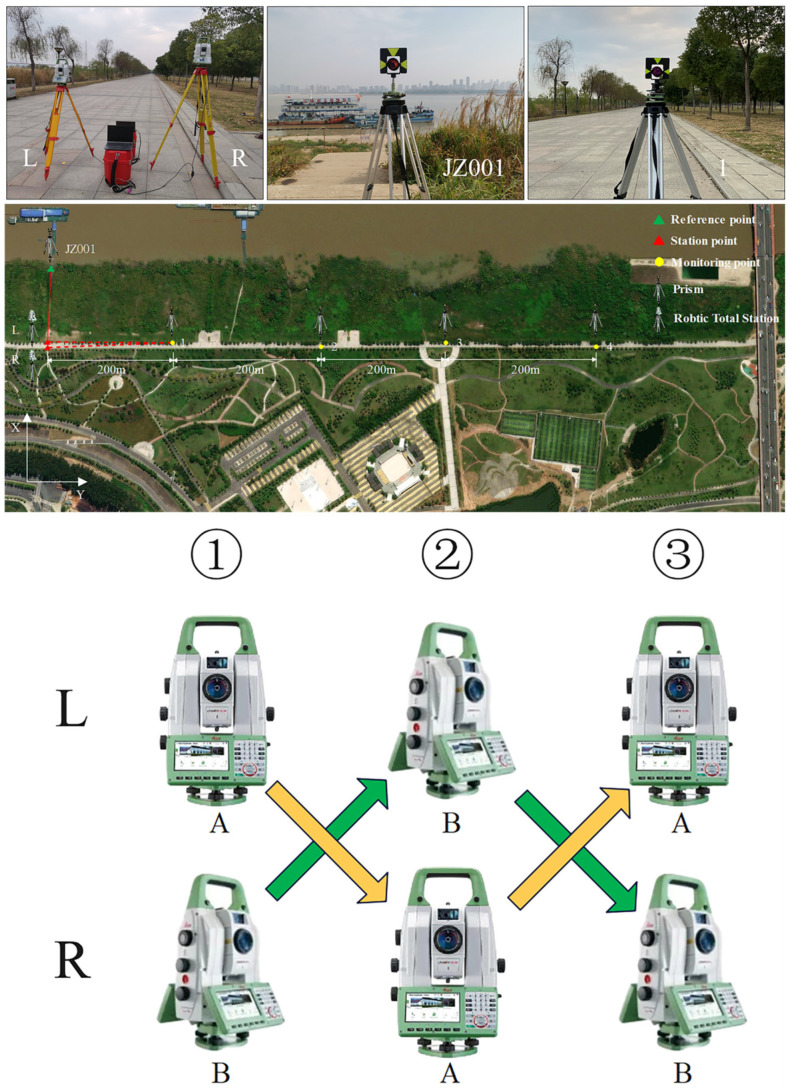
Feasibility experiments for the proposed method.

The primary experimental equipment consists of two Leica TS60 robotic total stations, denoted as A and B. They are equipped with accurate angular and distance measurement sensor systems, offering an angular accuracy of 0.5″ and a distance accuracy of 0.6 mm + 1 ppm within 3500 m. Two laptops equipped with automated monitoring software are connected to them to enable automated observation and data storage. The distribution of station points, reference points, and monitoring points in the experiment is shown in Figure 5. One station point is set at point R, and another station point L is placed at a distance of 2 m from R. This arrangement simulates the positions of station points at both ends of the fixed-length track. The direction defined by the line of the two station points is designated as the *X*-axis of the coordinates system. Consequently, the coordinates of the station points can be set as R (1000, 1000) and L (1002, 1000). The reference point JZ001 is positioned along the *X*-axis to serve as the initial direction for automated observation and calculation. Monitoring points are set at distances of approximately 200 m, 400 m, 600 m, and 800 m from the station points. All the monitoring points and the reference point are equipped with Leica GPR 121 circular prisms as the targets. The experimental procedure is as follows:

(1) Set up a robotic total station A at station point L and B at R. Utilize the automated monitoring software to obtain observations for each monitoring point and record them as 1LA and 1RB, respectively.
(2) Remove A and B from their tribraches and exchange their positions. In other words, place robotic total station A at station point R and B at L. Run another round of automated monitoring and record the observations as 2LB and 2RA.
(3) Once again, exchange the positions of robotic total station A and B and return to the setup in step (1). Conduct a new round of automated monitoring and record the observations as 3LA and 3RB.

**Figure 6:**
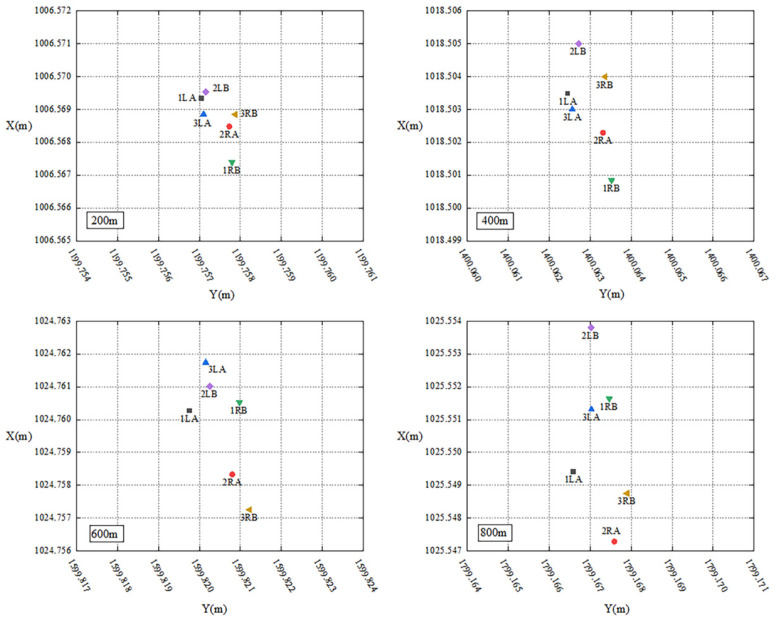
Coordinates distribution of polar coordinate in feasibility experiments.

For the impact of system errors from different instruments, observations from robotic total station A and B located at the same station point are utilized for analysis. Specifically, the coordinates of monitoring points calculated based on observations 1LA, 2LB, 3LA or 1RB, 2RA, 3RB are compared. According to the results shown in Figure 6, for station point L, the differences in coordinates in *Y* axis between robotic total station A and B align with the distance measurement accuracy of the robotic total station due to the *Y* axis being consistent with the distance measurement direction in the experiment. The maximum difference does not exceed 0.5 mm. However, the coordinates of monitoring points in the *X* axis are affected by both angular and distance observations, and there is a certain difference between robotic total station A and B, with a maximum difference of nearly 5 mm, exceeding the nominal accuracy of the instrument. According to the law of error propagation, the displacement error of monitoring points caused by the systematic error of different instruments can reach 7 mm. Similar results are observed at station point R with robotic total station A and B, which indicates the existence of a certain systematic error between the observations from different robotic total stations. Then, using two robotic total stations for the forward intersection will have a certain impact on the monitoring result in practical applications.

For the results of the polar coordinate system at different station points, observations of the same robotic total station located at station point L and R are selected for analysis. That is, the coordinates of monitoring points calculated from 1LA, 2RA, 3LA or 1RB, 2LB, 3RB are compared. According to the results in Figure 6, all the coordinates of monitoring points in *Y* axis calculated from station point R are slightly larger than those calculated from L. For the coordinates of monitoring points in *X* axis, there are differences between the results calculated from R station and L, which tend to increase with the monitoring distance. Similar results can be found in the coordinates of monitoring points calculated with a robotic total station B from station point L and R. It can be seen that there are certain differences in calculating the coordinates of the same monitoring point using the polar coordinate system under different station points, which is adverse to the unity of monitoring results.

**Figure 7:**
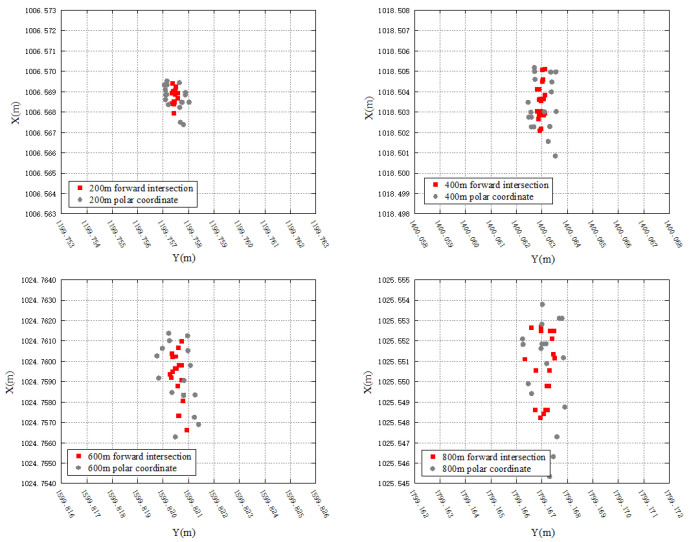
Comparison between polar coordinate and forward intersection.

Regarding the differences between the polar coordinate and forward intersection systems, the coordinates of monitoring points are calculated using both methods based on the observations obtained in the above experiments. The results are shown in Figure 7. Again, due to the *Y* axis being consistent with the distance measurement direction, the accuracy of coordinates in *Y* axis for monitoring points is better than that of the *X* axis for both the polar coordinate and forward intersection systems. However, regardless of the monitoring distance, the accuracy of forward intersection always outperforms that of polar coordinate system, which further confirms the reliability of the fixed-length track-assisted automated monitoring method using a single robotic total station.

**Figure 8:**
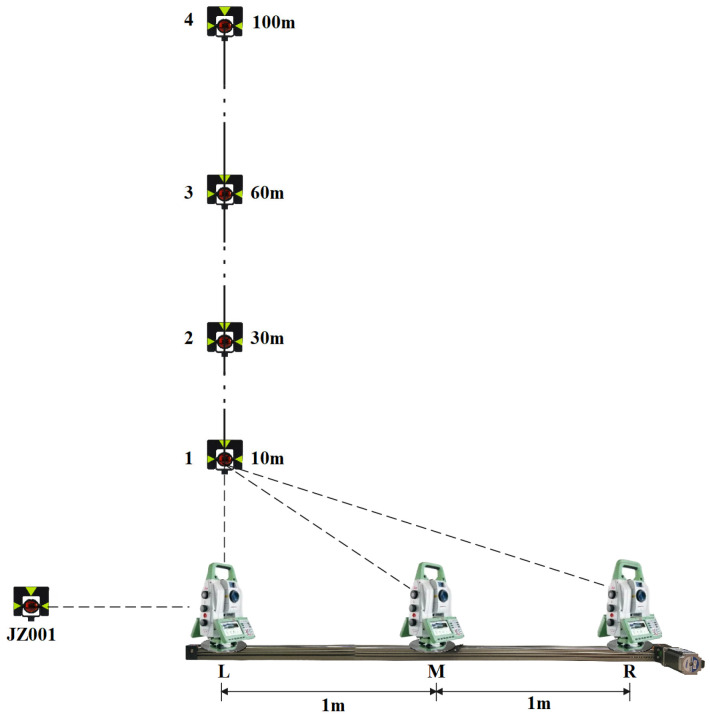
Experimental scheme when tracking the prism.

**Figure 9:**
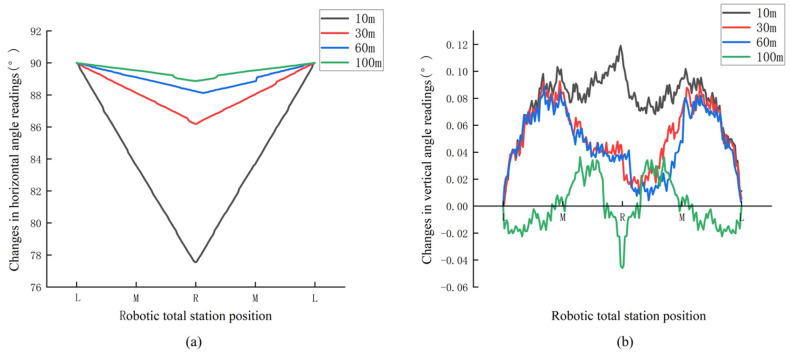
Changes in angle readings when tracking the prism: (**a**) horizontal angle; (**b**) vertical angle.

To ensure that the robotic total station can automatically locate and aim at the monitoring points represented by prisms to complete monitoring tasks while moving back and forth along the track, it is necessary to investigate the changes in angle readings during its movement on the track, which includes changes in horizontal and vertical angle readings depending on whether the robotic total station is capable of tracking the prism or not. When the robotic total station is capable of tracking the prism, the experimental scheme is depicted in Figure 8. A Leica TS60 robotic total station is set up on the track, where the distance between points L and R on the track is 2 m, with point M representing the midpoint, and a reference point JZ001 is set up in the LR line direction as the starting direction for observations. One Leica GPR 121 circular prism is placed at a distance of 10 m from point L in the vertical direction along the track. Initially, the robotic total station is placed at point L and locked onto the prism. Subsequently, the robotic total station moves smoothly from point L to R on the track and then returns to L with the help of the stepper motor. The system automatically records the horizontal and vertical angle readings of the robotic total station during the movement. Finally, the prism is placed at distances of 30 m, 60 m, and 100 m, respectively and the above process is repeated for each of these distances. Based on the outcomes presented in Figure 9, it is shown that the horizontal angle readings progressively decrease from 90° when the robotic total station moves from point L to R, reaching a minimum at point R and the readings increase gradually back to 90° when returning to point L. This observed trend is consistent with the results of theoretical analysis. Regarding the vertical angle readings, the observed changes when tracking the prisms also exhibit a certain degree of symmetry. The fluctuations in the vertical angle readings when tracking different prisms remain within a range of approximately 0.1°, which may be attributed to slight vibrations of the robotic total station during its movement along the track.

Additionally, by utilizing the precise geometric relationship between the robotic total station and the prisms, theoretical values for the horizontal angle readings at points M and R can be calculated. A comparison between the calculated theoretical and the observed readings of the robotic total station at these positions is presented in Table 1. It is shown that the difference between the theoretical and observed readings for the prism positioned at 10 m can reach approximately 1° due to the relatively short distance. For other distances, the maximum difference between theoretical and observed readings is around 8′. As a whole, the changes in horizontal and vertical angle readings of the robotic total station when moving along the track after tracking the prism are consistent with theoretical analysis. Therefore, when the robotic total station moves back and forth along the track, it can effectively achieve automatic locating and aiming at other prisms, with the tracked prism as a reference.

**Figure 10:**
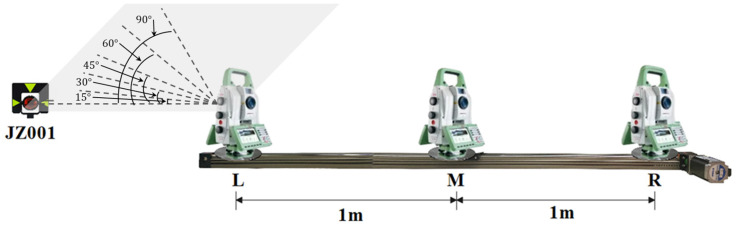
Experimental scheme without tracking the prism.

As some robotic total stations do not provide the function of tracking, the experimental scheme under such a situation is shown in Figure 10. The basic settings of the experiment are the same as before, except that there is no prism for tracking. Initially, the robotic total station is moved to point L and aimed at the reference point JZ001. The horizontal angle reading is set to 0°, and the vertical angle reading is adjusted to 90°. Following this, the robotic total station moves smoothly from point L to R and then returns to point L, driven by the stepper motor. During this process, stop the movement of the robotic total station at points L, M, and R for a while, and record the horizontal and vertical angle readings manually. Lastly, rotate the telescope of the robotic total station to make the horizontal angle readings as 15°, 30°, 45°, 60°, and 90°, while keeping the vertical angle reading as 90°. Then repeat the above experimental process.

**Figure 11:**
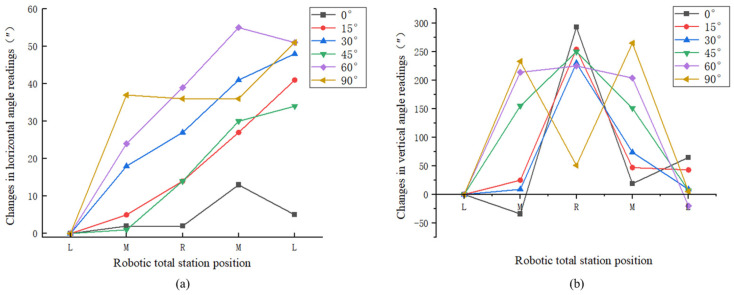
Changes in angle readings without tracking the prism: (**a**) horizontal angle; (**b**) vertical angle.

According to the results in Figure 11, when the telescope rotates to different directions, the changes in horizontal angle reading fluctuate randomly and remain within 1′ while the robotic total station moves back and forth on the track. The vertical angle readings change to a certain degree of symmetry and fluctuate within 5′. The main reason for this may be related to the smoothness of the track and the vibration during the movement of the robotic total station. The results indicate that the angle readings of the robotic total station remain almost stable when not tracking the prism. Based on the above findings and the automatic target recognition range of the robotic total station, it appears that the robotic total station can automatically locate and aim at the prisms when moving to both ends of the track even if it does not track a prism.

### 3.3. Practical Test

To further validate the advantages of the proposed method, a fixed-length track-assisted automated monitoring system is built to conduct the practical test. For the track system, the track adopts the RXP45 synchronous belt linear module with an effective travel of up to 2500 mm, which has a low friction coefficient when the slider platform moves back and forth. The motor adopts a 57-step motor, which can load 15 kg and has a positioning accuracy of 0.05 mm. The motor is driven by a 2D45M motor driver with a maximum pulse response frequency of 200 Kpps, which makes the current control smooth and accurate. Raspberry Pi 4B is used as the control terminal, and the track and robotic total station control software written in Python are stored in it for automated monitoring. The main instrument used for monitoring is the Leica TS60 robotic total station and a Leica GPR 121 circular prism is used as the monitoring target. To simulate the displacements in X and Y directions of the monitoring point, a three-axis translation stage is installed under the prism, which has a travel distance of 10 mm in all directions, with an accuracy of 10–20 μm. It can be used as a reference for measuring the displacements of monitoring points with robotic total station.

**Figure 12:**
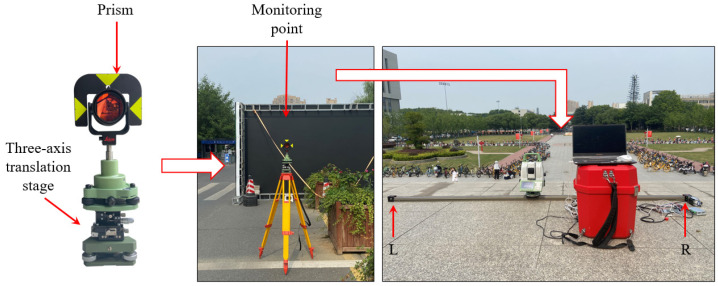
Practical test for the fixed-length track-assisted automated monitoring system.

The practical test took place in the morning in an open field. Throughout the test period, the weather was cloudy, with temperature of 32 °C, atmospheric pressure of 1013.30 hPa, and air humidity of 80%. As shown in Figure 12, the track system was put on the ground and adjusted to ensure that the robotic total station remained in a leveling state when moving to both ends of the track. Station points, labeled as L and R, were positioned at both ends of the 2 m track. The direction of LR line was designated as the *X* axis. A reference point was set on *X* axis and served as the starting direction for observation. At approximately 150 m and 300 m along the vertical direction of the track, prisms equipped with three-axis translation stage were installed as the monitoring points. The test processes are as follows:

(1) Manual training: Move the robotic total station to station point L and manually sight the reference point and monitoring points to record and store the training data. The approximate coordinates of monitoring points are then calculated and the training data for station point R are automatically calculated based on the spatial relationships between the robotic total station and monitoring points.
(2) Automated monitoring: Control the robotic total station to perform automated double-faced observation for the monitoring points located at 150 m and 300 m based on the manually acquired training data at station point L. After completing the observation at L, move the robotic total station to R, driven by the motor, without tracking the prism. Utilize the automatically calculated training data towards R to complete automated observation.
(3) Displacement simulation and observation: Use the three-axis translation stage to simulate the displacements of the monitoring points and carry out the automated monitoring with the robotic total station. Firstly, move the three-axis translation stages connected to the prisms along the *X* axis by 1 mm, and perform the automated monitoring in step (2) after each movement until the three-axis translation stages move 10 mm along the *X* axis. Then restore the three-axis translation stages to initial position and move them gradually along the *Y* axis by 1 mm until reaching their range of 10 mm. Similarly, perform the automated monitoring in step (2) for each movement.
(4) Data processing: Atmospheric corrections for the distances from each observation are executed according to the weather condition during the test. The coordinates of the monitoring points after each movement are calculated with the polar coordinate system firstly using the observations at L and R separately. Then, the observations at L and R are combined and the forward intersection is used to calculate the coordinates of the monitoring points after each movement.

**Figure 13:**
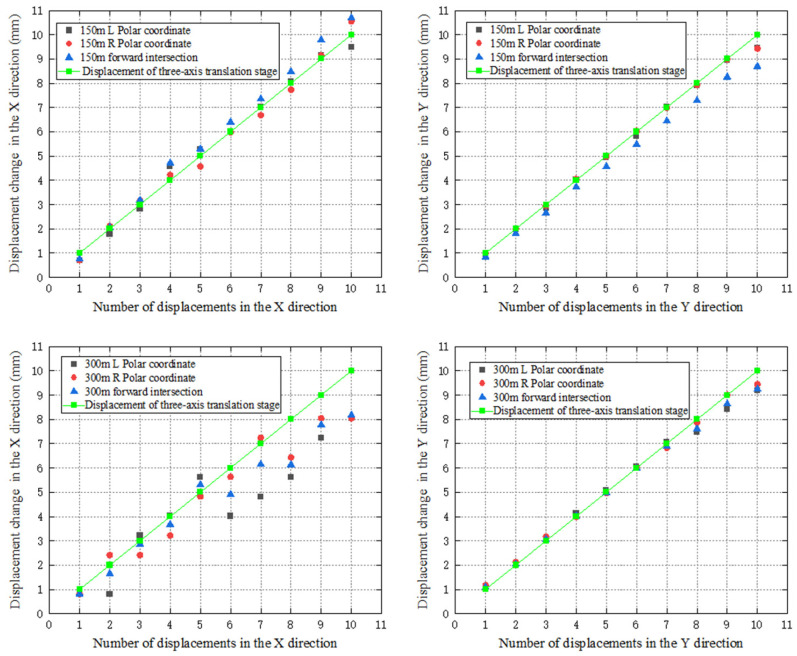
Results for the practical test.

The results are shown in Figure 13. It can be seen that for the displacements along the *X* axis, the results of the monitoring point at 150 m calculated using the polar coordinate and forward intersection systems are consistent with the results of the three-axis translation stage. For the monitoring point at 300 m, the difference between the results calculated using the polar coordinate system and the three-axis translation stage can reach a maximum of 2 mm. Although the results calculated using forward intersection have some differences compared with the three-axis translation stage, it is, overall, better than that of the polar coordinate system. For the displacements along the *Y* axis, the results of the monitoring points at 150 m and 300 m using both polar coordinate and forward intersection systems are consistent with the three-axis translation stage.

Furthermore, the displacements in the X and Y axes calculated by the robotic total station through different methods are subtracted from the reference values provided by the three-axis translation stage, and the standard deviations are calculated as shown in Table 2. According to Table 2, the displacement standard deviation in the *X* axis for the forward intersection system becomes smaller than that of the polar coordinate when the monitoring distance increases from 150 m to 300 m. For the *Y* axis, the displacement standard deviation of forward intersection has no advantage over the polar coordinate system, mainly because the *Y* axis in the test is consistent with the distance measurement direction, so the displacement is almost affected by the distance measurement accuracy only.

The practical test verifies that, as the monitoring distance increases, the reliability of the monitoring results obtained with the proposed method is better than that of polar coordinate system, where observations from only one station point are utilized. It can be expected that when faced with interference from complex environmental factors in actual monitoring tasks, the proposed method can enhance the reliability of the monitoring results as redundant observations are provided.

## 4. Conclusions

This study addresses the shortcomings of polar coordinate and forward intersection systems commonly used in automated monitoring with robotic total station, and proposes an automated monitoring method with forward intersection implemented by a single robotic total station assisted by a fixed-length track. Simulation results show that even with a small distance between the station points, the forward intersection method can achieve better accuracy than that of the polar coordinate method. Feasibility tests show that systematic error between different robotic total stations can reach approximately 5 mm. The comparison between polar coordinate and forward intersection methods confirms the advantages in accuracy for the proposed method and the changes in the horizontal and vertical angle readings when the robotic total station moves on the track meet theoretical expectations, which ensures that the robotic total station can automatically locate and sight the prisms while moving back and forth on the track and complete automated monitoring. Practical test results indicate that as the monitoring distance increases, the reliability of the monitoring results for the proposed method is better than that of polar coordinate system.

Compared to existing monitoring methods based on forward intersection, the proposed method achieves excellent cost savings as only one robotic total station is required and improves automation. Currently, the proposed method has achieved good results in laboratory and field tests, but lacks further verification in practical engineering applications. It is expected that in the future, practical monitoring projects can be conducted to verify the reliability of the proposed method.

## Figures and Tables

**Table 1:** Comparison between the theoretical and observed horizontal angle readings.

Positions on Track (m)	Monitoring Distance (m)	Theoretical Horizontal Angle Readings	Observed Horizontal Angle Readings	Differences
M	10	84°17′21.9″	83°28′55.0″	0°48′26.9″
M	30	88°5′27.1″	88°6′55.0″	0°1′28.0″
M	60	89°2′42.6″	89°6′03.0″	0°3′20.4″
M	100	89°25′37.4″	89°31′56.5″	0°6′19.1″
R	10	78°41′24.2″	77°32′41.1″	1°8′43.1″
R	30	86°11′09.3″	86°11′08.4″	0°0′01.0″
R	60	88°5′27.1″	88°13′55.0″	0°8′27.9″
R	100	88°51′15.3″	88°52′00.0″	0°0′44.8″

**Table 2:** Displacements standard deviations with different methods.

Direction of Displacement	X			Y		
Monitoring Methods	Polar Coordinate at L	Polar Coordinate at R	Forward Intersection	Polar Coordinate at L	Polar Coordinate at R	Forward Intersection
Standard deviation at 150 m(mm)	0.29	0.29	0.30	0.15	0.17	0.19
Standard deviation at 300 m(mm)	1.08	0.81	0.59	0.32	0.20	0.26

## Data Availability

The data supporting the findings of this study are available within the article.
